# Surgery in Rheumatic Fever

**Published:** 1903-02-07

**Authors:** 


### SURGERY IN RHEUMATIC FEVER.

Much has been said of late about septic arthritis and the treatment of certain forms of the disease by free incision. But if it should turn out to be the fact as is suspected by so many that rheumatic fever is caused by a micro-organism, and that the joint effusions met with in that disease are themselves toxic as is the case in those forms of arthritis which are now generally recognised as septic, the question would seriously arise whether the same kind of treatment, namely by free incision and drainage, which has been found necessary in the one class of cases would not be properly applicable in the other. As bearing upon this question the remarks made by Dr. John O'Conor,[1] of Buenos Ayres, on the surgical treatment of rheumatic fever are of much importance, especially as his conclusions are supported by a series of 20 cases in which the treatment above referred to was carried out. He admits that he has met with non-success in certain chronic cases, particularly in those of a dry variety, the result of a prolonged pickling of the synovial membrane in infective material, but says that in the condition commonly understood as rheumatic fever, acute rheumatic arthritis, or acute rheumatism, he is certain that surgical treatment is most efficacious, not only in causing immediate disappearance of the arthritis and early cessation of the general toxæmia, but also in effectually protecting the heart against attack. Believing as he does that rheumatic fever is an infective disease, and that the infected joints serve as incubators where the poison is elaborated and whence it is passed into the circulation and thus conveyed to other joints and to the heart, he maintains that removal of the infective material as soon as possible from the infected joints is a scientific and rational method of treatment, a conclusion which he says is fully corroborated by results. Certainly the cases which he relates go far to support his views. In some of them the knees were opened, in others the ankles or wrists; but Dr. O'Conor points out that in some cases a form of infective watery cellulitis appears to be the dominant manifestation of the disease about the ankle and wrist joints, and that in these cases multiple incisions into this effusion are required. Of course every precaution must be taken to ensure full asepsis in the operation and after-treatment. The knees are to be freely opened by an incision an inch long through the capsule on each side, thoroughly drained by means of a fenestrated drainage tube half an inch in diameter passed right through, and irrigated daily with a jugful of warm carbolic lotion (1 in 60). The tube is generally dispensed with on the third or fourth day. In dealing with the wrist and ankle-joints a gauze drain is employed.

[1] Lancet, Jan. 24.

